# Gains in life expectancy in the Australian population due to reductions in smoking: comparisons between interventions targeting the population versus interventions in a specific high risk group

**DOI:** 10.1186/s12889-020-09600-w

**Published:** 2020-09-29

**Authors:** Haider Mannan

**Affiliations:** Translational Health Research Institute, Western Sydney University, Campbelltown, NSW 2560 Australia

**Keywords:** Smoking reduction, Population approach, High risk approach, Combined approach, Average life expectancy, Risk percentiles method

## Abstract

**Background:**

Four decades of population-based tobacco control strategies have contributed to substantial reduction in smoking prevalence in Australia. However, smoking prevalence is still double in socially disadvantaged groups compared to those that are not. But not all tobacco control strategies successfully used in the general population is effective in specific high-risk population groups. Hence, an effective way to reduce smoking in high risk population groups may include targeting them specifically to identify and support smokers to quit. In this backdrop, we examined whether tobacco control interventions at the population-level are more effective in increasing life expectancy among Australians compared to interventions targeting a high risk group or a combination of the two when smoking prevalence is reduced to 10 and 0% respectively.

**Methods:**

Using the risk percentiles approach, analyses were performed separately for men and women using data from various sources such as the 2014–15 National Health Survey linked to death registry, simulated data for high risk groups, and the Australian population and deaths data from the census. Indigenous status was simulated by preferentially assigning those who are indigenous to lower SES quintiles. The age-sex distribution of mental disorder status was simulated using its distribution from 2016 National Drug Strategy Household Survey with 25.9% of mentally ill being assigned to current smoking category and the rest to non-smoking category. The age-sex distribution of prisoners was simulated based on 2014 ABS Prisoners Australia survey with 74% of prisoners being assigned to current smoker category and the rest to non-smoker category. Homelessness status was simulated according to age, sex and indigenous status for 2011 census with all homeless being allocated to the lowest SES category. The age-sex distribution of total cholesterol level was simulated based on 2011–13 Australian Health Survey.

**Results:**

The results showed that the combined approach for reducing smoking is most effective for improving life expectancy of Australians particularly for the socially disadvantaged and mentally ill groups both of which have high fraction of smokers in the population. For those who were mentally ill the gain in ALE due to reduction of smoking to 10% was 0.53 years for males and 0.36 years for females which were around 51 and 42% respectively of the maximal gains in ALE that could be achieved through complete cessation.

**Conclusions:**

Targeting high-risk population groups having substantial fraction of smokers in the population can strongly complement the existing population-based smoking reduction strategies. As population and high risk approaches are both important, the national prevention policies should make judicious use of both to maximize health gain.

## Background

Tobacco smoking is a key risk factor of the four diseases, namely, coronary heart disease, lung cancer, stroke and coronary obstructive pulmonary disease (COPD) that cause most deaths in Australia. It is the single most important preventable cause of ill health and death according to the Australian Burden of Disease study [1], despite the fact that its prevalence in the Australian population has been declining since the 1950s, and Australia currently has one of the lowest rates of smoking in the developed world, with a prevalence of current daily smokers of 14.5% reported in 2014–15 among adults aged 18 years and above [2]. Among risk factors, smoking caused the most burden of disease in 2015 with an estimated 77.7% of lung cancer, 72.4% of COPD and 40.3% of cardiovascular disease (CVD) burdens being attributable to smoking [1]. For prior to age 65 and all ages 38 and 16% of CVD deaths are attributable to smoking [3]. Moreover, smoking contributes to more drug-related hospitalisations and deaths than alcohol and illicit drug use combined [4]. It is therefore a major contributor to the costs of the health system. In addition to the direct costs associated with provision of care for smoking-related illness, additional costs to the community include loss of productivity due to absenteeism and reduction in the workforce resulting from premature death.

Australia’s declining smoking prevalence is largely due to the collective effects of the numerous population-based tobacco control strategies introduced over the past four decades. Smoking has declined in all socioeconomic groups over time with the relative gap in smoking prevalence between the most and least disadvantaged groups narrowing between 2013 and 2018 [5–8]. which is because smoking has declined more rapidly among disadvantaged smokers [7, 8]. However, smoking rates are still double in least advantaged groups compared to the most advantaged groups [6]. Because of this, one of the key priority areas of Australia’s 2012–18 National Tobacco Strategy is strengthening efforts to reduce smoking among groups with a high prevalence of smoking [9]. However, tobacco control measures effectively used in the general population may not always be effective in specific high-risk populations, eg., a meta-analysis of smoking cessation programs in highly disadvantaged groups cited the lack of high-quality evidence among the homeless, indigenous and prisoners [10, 11]. Therefore, apart from population-based strategies, another way for reducing smoking in high risk population groups may be to specifically target these groups to more effectively identify and support those smoking to quit (a.k.a high risk approach).

It is unknown whether the population or high risk approach or a combination of the two, for reducing smoking is more effective in prolonging life expectancy in the Australian population. Accordingly, the aim of this paper is to compare the relative effectiveness of these three basic approaches for reducing smoking in a population. For our analysis the smoking prevalence is reduced to 0 and 10% from its existing level, as various Australian government institutions have come to a consensus in their aim to reduce the national smoking prevalence to 10% by 2020 [12]. All analyses in this study are performed separately for men and women. The high risk population subgroups examined are socioeconomically disadvantaged, prisoners, indigenous, homeless and mentally ill, as these are among the major population subgroups having high smoking prevalence [13]. Drug use is not considered as a high risk group because smoking prevalence was only around 16% among drug users in 2010 [14]. while remoteness is not considered because rural and remote regions of Australia are already more likely to be of lower socioeconomic status [15].

### Impact of key population-based smoking reduction strategies on population subgroups of varying risks

The major population based approaches in context of Australia include increase of tobacco taxes, bans on advertising, public health campaigns, smoke free legislation, jurisdictional bans and limitations on point-of-sale display, mandatory graphic health warnings on packaging and the introduction of plain packaging. For almost two decades tobacco excise was indexed to inflation, until 2010, when there was a one-off increase in excise to 25% with four subsequent annual increases of 12.5% from 2013 to 2016. That has seen cigarette prices increase from about 15 dollars a pack in 2010, to around 25–30 dollars in 2016.

It has been noted that an important consideration in the development of public health policy in Australia is whether tobacco control strategies are as effective in reaching high risk groups (e.g., low SES) as they are in reducing smoking among normal or low risk groups (e.g., high SES) [16–19]. During a period of strong tobacco control activity from 1997 till 2005 in Australia [8], and from 1997 till 2011 in Victoria [7], smoking declined across all population subgroups but it was fastest in high risk groups (eg., low SES groups) among teenagers aged 12–15 years and adults respectively. Specifically, for a large number of population-based tobacco control strategies like the 2010 tax increase on tobacco products [20, 21], higher merchant compliance with regulations on the supply of tobacco products [22], introduction to smoke-free hospitality venues [23] and anti-smoking mass media campaigns [23], smoking prevalence has declined fastest among high risk population subgroups while for higher merchant compliance [22], graphic warnings on packages [24], 2010 tax increase [25] and anti-smoking mass media campaigns [25] overall smoking prevalence has also declined, however, for 2010 tax increase the decline in smoking did not sustain after 3 months [26]. The faster decline of smoking as seen in high risk population groups is non-existent for the use of mass media [27] while there is no comparable data for introduction to plain packaging or graphic warnings on packages.

### Smoking rate in the selected high risk groups

The smoking prevalence in 2014–15 National Health Survey (NHS) was 14.5% overall and 16.9% for males and 12.1% for females [28]. There were 21.4% of people who were most disadvantaged (first quintile) and smoked daily, compared with 8.0% of people who were the least disadvantaged (fifth quintile). Rates of smoking have decreased over the past decade in all quintiles of disadvantage. However, the daily smoking rate of 21.4% for the most disadvantaged is still the highest among all the disadvantaged groups.

The mentally ill represented 17.5% of the Australian adult population in 2016 [29]. Data from the 2016 National Drug Strategy Household Survey also showed that Australian adults who reported having been diagnosed or treated for mental illness in the past year had 25.9% smoking rate which was more than twice as likely than that of 12.3% for those who were not diagnosed or treated in the past year [29].

Although smoking prevalence among prisoners (74%) [30] and homeless (77%) [13] was even higher than among mentally ill (25.9%) and those having low socioeconomic status (SES) (21.4%), they represented a much smaller fraction of smoking population as the prisoners and homeless both represented less than 1% of the total population in Australia in 2014 [31, 13]. The indigenous had a 41.85% rate of daily smoking in 2014–15 [32] which was much lower than the prisoners and homeless, but they represented a small fraction (around 1.7%) of smoking population similar but slightly larger than the prisoners and homeless.

### Effectiveness of population-based approach versus high risk approach for preventing disease

Rose (2001) proposed that preventing disease by trying to shift the whole population distribution of a risk factor can be more efficient than focusing interventions solely on people at high risk [33]. This occurs when a disease is widespread and many cases of the disease arise in individuals who are not in a high-risk group, and the number of cases arising from the population at average risk is often greater than the number occurring in the population at high risk simply because there are so many more people in the average-risk population. However, when many cases of disease arise in individuals who are in a high-risk group such disease is not compliant to the population disease prevention strategy and hence it is possible that for a certain disease the high risk approach can be more efficient. The optimum preventive strategy therefore depends on the disease to be prevented, the distribution of its risk factors in the population and the likelihood of achieving the desired reduction in the risk factor.

## Methods

### Data sources

The primary data source is 2014–15 NHS which is a nationally representative survey in Australia having detailed information on health risk factors, socioeconomic and demographic characteristics and health services. It is the most recent in a series of Australia-wide health surveys conducted by the Australian Bureau of Statistics and was conducted in all states and territories and across urban, rural and remote areas of Australia (other than very remote areas) from July 2014 to June 2015. The other data sources include the 2011 Australian census, 2011–13 Australian Health Survey (AHS), 2016 National Drug Strategy Household Survey, ABS Prisoners Australia 2014 and Australian census-based population and death counts for 2015–17.

### Ethics

The study was not required to be submitted and approved by the institutional ethics committee of Western Sydney University where the author is based. The need for ethics approval was waived by an IRB or was deemed unnecessary according to national regulations, with the name of the IRB or a reference to the relevant legislation being EX2020–13.

### Simulation methodology

Using the 2014–15 NHS sample a random reduction in smoking prevalence to 10 and 0% was simulated for the whole sample as well as for a specific high risk group and a combination of the two. First, a random general reduction, where the simulated reduction was randomly selected in the whole sample, with selected individuals becoming non-smokers. This was labelled the ‘population approach’. The second approach, labelled the “high risk approach”, was designed to target a specific high risk group (i.e. prisoners, homeless, mentally ill, indigenous, low SES), with the prevalence reduction occurring only in a specific group.

Finally, a combination of the specific approach with the population approach, labelled the “combined approach”, was designed to target a specific group as well as the whole sample. Each smoking prevalence reduction scenario and combined scenario was simulated independently of each other.

Using the 2014–15 NHS linked to ABS’s deaths registry till 2017 the current smoking status of individuals was assigned while their SES was assigned according to the 2011 Index of Relative Socioeconomic Disadvantage (SEIFA) variable available in the same linked dataset. However, the variables which were either not available (eg., indigenous status, prisoner status, homelessness status, total cholesterol level) or underestimated (eg., mental disorder status) in the linked 2014–15 NHS were simulated. For example, indigenous status was simulated by preferentially assigning those who are indigenous to lower SES quintiles, using the following distribution: 32.3, 29.3, 19.7, 11.3 and 7.4%. This is an approximation of the distribution found on ABS’s 2011 census [34]. Among indigenous males, 32.2% were current smokers while among indigenous females 31.15% were current smokers. Of those males belonging to the lowest SES quintile, 92.85% were current smokers while for females belonging to this group, 85.18% were current smokers.

**Fig. 1:**
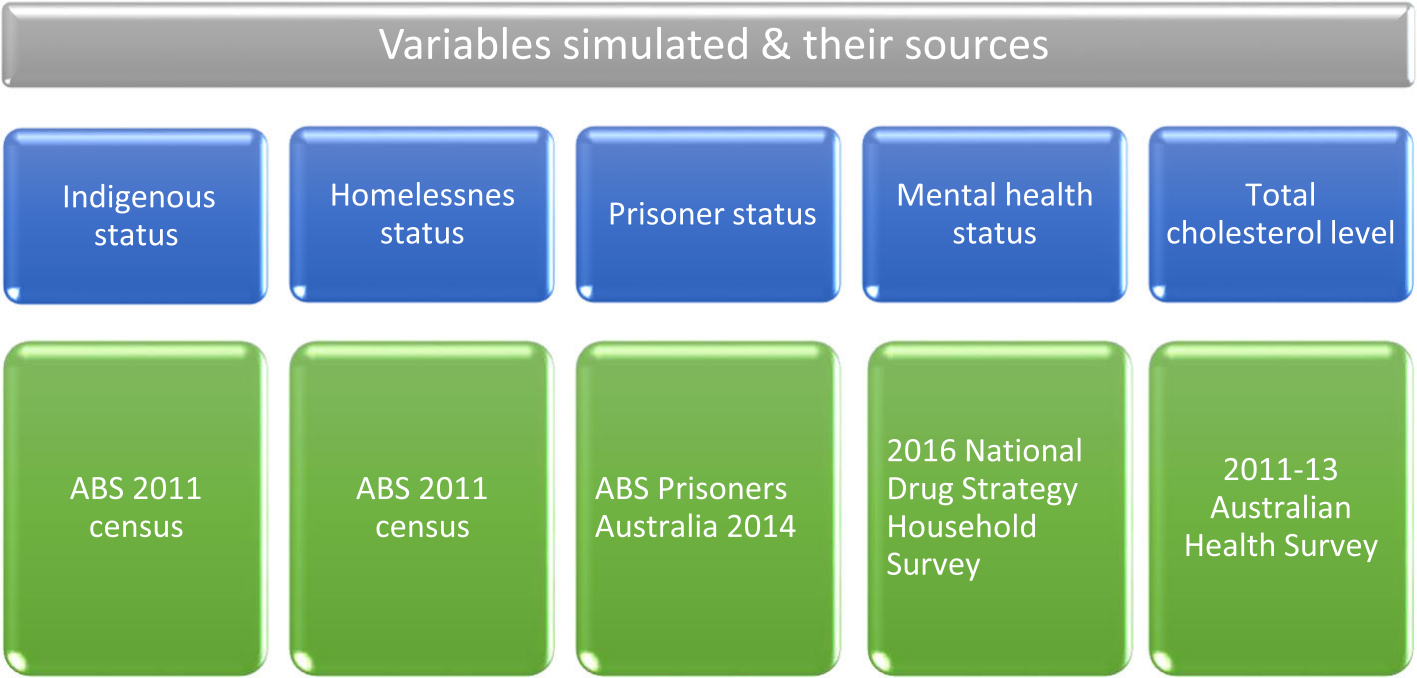
Flow chart of variables simulated and their sources

The age-sex distribution of mental disorder status among the individuals was simulated using the age and sex distribution of those who are mentally ill according to the 2016 National Drug Strategy Household Survey having an overall prevalence of 17.5% [29]. 25.9% of those mentally ill were assigned to current smoker category and the rest 74.1% to non-smoker category. For mentally ill males 85.74% were current smokers while for mentally ill females 70.4% were current smokers. The distribution of prisoners according to age and sex in Australian Bureau of Statistics (ABS) Prisoners Australia 2014 was used to simulate prisoner status having an overall prevalence of 0.1856% [31]. 74% of the prisoners were assigned to current smoker category and the rest 26% to non-smoker category. Homelessness status (HL) was attributed according to age, sex and indigenous status (IS) for 2011 census [34]. All homeless individuals were allocated to the lowest SES category. For the homeless males, 9.7% were smokers while for homeless females, 8.5% were smokers. The age-sex distribution of the risk factor total cholesterol level was simulated using its age and sex distribution from the 2011–13 AHS [35] which has a new collection called the biomedical measures component where total cholesterol was measured by blood test. Figure 1 below shows the variables simulated and their data sources.

In our scenario modelling to reduce the overall smoking prevalence randomly to 10%, the smoking status of some individuals in the sample who were current smokers was randomly changed to “non-smoker” by sorting the distribution of current smoking by a random variable generated from continuous uniform distribution. On the contrary, the smoking status of non-smokers was not changed. For a person whose status changed from smoker to non-smoker, we then obtained a reduced risk of mortality and smoking prevalence compared to the data on smoking prevalence we simulated as described above. The random selection process was replicated 1000 times and the study outputs, such as, ALEs as presented in this article, are all averages over the 1000 replications. An identical method was performed to achieve a smoking prevalence of 10% under a specific high risk or combined scenario (e.g. combined population, homeless, indigenous, mentally ill, lowest SES group), by randomly selecting some of the current smokers belonging to a relevant group or groups to have their smoking status changed to non-smoking.

### External validation of the EURO SCORE to the 2014–15 NHS data

The age, total cholesterol and systolic blood pressure variables from 2014 to 15 NHS were used to estimate the absolute risk of all-cause death separately for males and females aged 15 and above using the EURO SCORE risk equations. To assess the suitability of the SCORE statistic as a proxy for all-cause mortality risk in this study, we used the 2014–15 NHS sample linked prospectively to ABS’s Australian death registry data for 2 years till 2017. We used Cox proportional hazards regression separately for males and females for predicting the risk of all-cause death with the SCORE statistic and age (continuous) as predictors. As the SCORE statistic was developed to allocate short-term mortality, we chose a follow-up of 2 years in our study. Age was included alongside SCORE as predictors because the EURO SCORE equation was used in this study on a sample aged 15 and above while it was originally developed for a European cohort aged 45–64. We also fitted Cox regression to ages 65 and over to demonstrate the predictive and discriminatory abilities of SCORE at older ages. The regression equations’ fit and discriminatory abilities were assessed using the Hosmer-Lemeshow and Harrell’s C statistics [36, 37]. The external validation methodology for the EURO SCORE equation as discussed above is similar to the ones used in our previous studies [38, 39].

### Risk percentiles method

The risk percentiles method was used for estimating the average life expectancy (ALE) within the simulated Australian population or subgroups. An earlier coronary heart disease (CHD) prevention model was modified to obtain this method [40]. Details of this method and the calculations of ALE are provided in Appendix (check ‘Additional file 1.docx’). Briefly, the steps required are:

- Use the EURO SCORE to estimate risk scores for mortality for every individual in our study cohort.
- Using these risk scores divide the cohort into mortality risk percentiles.
- Estimate the ratios of risk scores for smokers relative to non-smokers and use them to proportionately allocate ABS death counts to each sex and age group specific risk percentiles in the Australian population from 2015 to 2017.
- Within each sex and age group divide these death counts by the sex and age group-specific Australian population to give us the mortality rate for each risk percentile group.
- Using these mortality rates construct sex-specific life tables for each risk percentile within each sex group.
- For each sex calculate a baseline ALE per person by averaging the life expectancy associated with each risk level over the population.
- Construct 95% confidence interval for ALE using bootstrapping.
- In order to achieve the desired target of 10% smoking prevalence change a certain percentage of current smokers to non-smokers.
- Participants are then re-allocated to risk percentiles.
- Calculate the scenario ALE by averaging the life expectancy associated with each re-allocated risk level over the population.
- Construct 95% confidence interval for the scenario ALE using bootstrapping.
- Calculate the gain in ALE by subtracting the average life expectancy for the “Base Scenario” from that for each intervention.

For each sex a total of 23 scenarios were simulated as shown in Table 1.

**Table 1:** Description of the simulated scenarios

Scenario	Targeted Population	Change in smoking behavior
**Base scenario**	Whole population	No change in baseline smoking prevalence for both men and women
**10% prevalence in Whole population**	Whole population	Prevalence randomly reduced to 10% for both men and women
**0% prevalence in Whole population**	Whole population	All smokers in targeted group quit
**10% prevalence in homeless**	Homeless Group	Prevalence randomly reduced to 10% for both men and women who are homeless
**10% prevalence in both population & homeless**	Whole population & homeless Group	Prevalence randomly reduced to 10% for both men and women in targeted groups
**0% prevalence in homeless**	Homeless Group	All smokers in targeted group quit
**0% prevalence in both population & homeless**	Whole population & homeless Group	Prevalence reduced to 10% for both men and women in targeted groups
**10% prevalence in drug users**	Drug users Group	Prevalence randomly reduced to 10% for both men and women who are drug users
**10% prevalence in both population & drug users**	Whole population & drug users Group	Prevalence randomly reduced to 10% for both men and women in targeted groups
**0% prevalence in drug users**	Drug users Group	All smokers in targeted group quit
**0% prevalence in both population & drug users**	Whole population & drug users Group	All smokers in targeted groups quit
**10% prevalence in mentally ill**	Mentally ill Group	Prevalence randomly reduced to 10% for both men and women who are mentally ill
**10% prevalence in both population & mentally ill**	Whole population & mentally ill group	Prevalence randomly reduced to 10% for both men and women in targeted groups
**0% prevalence in mentally ill**	Mentally ill Group	All smokers in targeted group quit
**0% prevalence in both population & mentally ill**	Whole population & mentally ill group	All smokers in targeted groups quit
**10% prevalence in low SES**	Low SES Group	Prevalence randomly reduced to 10% for both men and women who belong to low SES group
**10% prevalence in both population & low SES group**	Whole population & low SES group	Prevalence randomly reduced to 10% for both men and women in targeted groups
**0% prevalence in low SES group**	Low SES Group	All smokers in targeted group quit
**0% prevalence in both population & low SES group**	Whole population & low SES Group	All smokers in targeted groups quit
**10% prevalence in Indigenous group**	Indigenous Group	Prevalence randomly reduced to 10% for both men and women who are Indigenous
**10% prevalence in both population & Indigenous group**	Whole population & Indigenous group	Prevalence randomly reduced to 10% for both men and women in targeted groups
**0% prevalence in Indigenous group**	Indigenous Group	All smokers in targeted group quit
**0% prevalence in both population & Indigenous group**	Whole population & Indigenous group	All smokers in targeted groups quit

We simulated a total of 1000 replications. The software SAS version 9.4 was used to perform all simulations and analyses [41].

## Results

### Sensitivity analysis

Although SCORE was originally derived to predict risk at ages 45–64, it was applied to ages 15 and over in this study. So to demonstrate SCORE’s overall ability to predict all-cause mortality risk to ages 15 and over we fitted the Cox regression and also fitted a separate regression to ages 65 and over to demonstrate its predictive ability at older ages. There were 283 deaths prior to 1 June 2017 among the 18,287 records linked to the outcome. For both men and women, the EURO SCORE index was a strongly significant predictor of mortality for ages 15 and over (results not shown). The adjusted Hosmer-Lemeshow goodness of fit statistic showed no evidence of lack of fit for both males and females (adj HL (df) = 7.70 (9), *p* = 0.564 for males; adj HL (df) = 7.41 (9), *p* = 0.594 for females) and the C statistics had values of 86.8% for men and 90% for women [42] where C values above 80% are generally regarded as demonstrating excellent discriminatory power [43]. The SCORE index was still a strong predictor (*p* < 0.0001) of mortality for either males or females when the regression is restricted to ages 65 and over while the adjusted Hosmer-Lemeshow goodness of fit statistic showed no evidence of lack of fit in either equation and the C statistics showed reasonable discriminatory power with values of 74% for men and 74.5% for women.

### Results of scenario analysis

A gain in ALE associated with the reduction of smoking prevalence was observed using all the three approaches [44]. This is shown in Table**s** 2 and 3. The gain in average life expectancy using the population approach was approximately 0.31 years for males and 0.18 years for females when smoking prevalence was reduced to 10%. These gains were 40 and 27% of the maximal gains in ALE that could be achieved through complete cessation for which the gain was 0.78 years for males and 0.67 years for females.

**Table 2:** Gain in average life expectancy in the population under various smoking reduction scenarios for males

Scenarios	ALE (95% CI)	Gain in ALE relative to Base scenario
Baseline	36.12 (35.81, 36.43)	–
10% rate	36.43 (35.89, 36.88)	0.31
0% rate	36.90 (36.33, 37.63)	0.78
10% rate Homeless	36.17 (35.69, 36.78)	0.05
10% rate pop & homeless	36.48 (35.86, 37.03)	0.36
0% rate homeless	36.18 (35.65, 36.59)	0.06
0% rate pop & homeless	36.95 (36.34, 37.39)	0.83
10% rate prisoner	36.17 (35.19, 36.82)	0.05
10% rate pop & prisoner	36.51 (35.88, 37.01)	0.39
0% rate prisoner	36.38 (35.85, 36.86)	0.26
0% rate pop & prisoner	37.02 (36.41, 37.47)	0.90
10% rate mentally ill	36.40 (36.04, 37.25)	0.28
10% rate pop & mentally ill	36.65 (36.23,37.61)	0.53
0% rate mentally ill	36.52 (36.49, 38.52)	0.40
0% rate pop & mentally ill	37.15 (36.63, 37.89)	1.03
10% rate indigenous	36.20 (35.12,36.85)	0.08
10% rate pop & Indigenous	36.51 (35.92, 37.17)	0.39
0% rate indigenous	36.21 (35.74, 37.78)	0.09
0% rate pop & indigenous	36.99 (36.33, 37.85)	0.87
10% rate low SES	36.35 (35.90, 37.30)	0.23
10% rate pop & low SES	36.60 (36.05, 37.50)	0.48
0% rate low SES	36.44 (36.00, 37.14)	0.32
0% rate pop & low SES	37.10 (36.47, 38.02)	0.98

**Table 3:** Gain in average life expectancy in the population under various smoking reduction scenarios for females

Scenarios	ALE (95% CI)	Gain in ALE relative to Base scenario
Baseline	40.21 (39.87, 40.55)	–
10% rate	40.39 (40.00, 40.97)	0.18
0% rate	40.88 (40.25, 41.37)	0.67
10% rate Homeless	40.27 (39.42, 40.89)	0.06
10% rate pop & homeless	40.52 (39.88, 41.02)	0.31
0% rate homeless	40.31 (39.61, 40.58)	0.10
0% rate pop & homeless	41.03 (40.22, 41.48)	0.82
10% rate prisoner	40.25 (39.70, 40.65)	0.04
10% rate pop & homeless	40.48 (39.97,40.88)	0.27
0% rate prisoner	40.33 (39.83, 40.38)	0.12
0% rate pop & prisoner	40.97 (40.08, 41.58)	0.76
10% rate mentally ill	40.42 (39.92, 40.88)	0.21
10% rate pop & mentally ill	40.58 (39.98, 41.30)	0.36
0% rate mentally ill	40.50 (40.10, 40.97)	0.29
0% rate pop & mentally ill	41.06 (40.63, 41.57)	0.85
10% rate indigenous	40.32 (39.86,40.79)	0.11
10% rate pop & Indigenous	40.50 (40.04, 41.23)	0.29
0% rate indigenous	40.37 (39.88, 40.93)	0.16
0% rate pop & indigenous	41.04 (40.40, 41.86)	0.83
10% rate low SES	40.38 (39.92, 41.08)	0.17
10% rate pop & low SES	40.52 (39.94, 41.13)	0.31
0% rate low SES	40.49 (40.02, 41.51)	0.28
0% rate pop & low SES	41.09 (40.54, 41.83)	0.88

The gains using the high risk approach were very low in all specific subgroups, except among mentally ill populations. For reductions to 10% smoking prevalence, the gain in average life expectancy when the mentally ill were targeted was 0.28 years for males and 0.21 years for females. For complete smoking cessation, the gain in average life expectancy when the mentally ill were targeted was 0.40 years for males and 0.29 years for females. For reductions to 10% smoking prevalence, the gain in average life expectancy when people in the lowest socioeconomic quintile were targeted was 0.23 years for males and 0.17 years for females. For complete smoking cessation, the gain in average life expectancy when people in the lowest socioeconomic quintile were targeted was 0.36 years for males and 0.28 years for females. Both the lowest socioeconomic quintile and mentally ill subgroups have substantial fraction of smokers in the population as 20 and 17.5% of the population belongs to these subgroups respectively. On the contrary, the indigenous, homeless and prisoner subgroups have much smaller fraction of smokers in the population as only 1.7, 0.489 and 0.1856% of the population belongs to these subgroups respectively.

The gains in ALEs for males and females using the combined approach compared to the high risk approach were substantially higher. The combined approach produced also higher gains in ALE compared to the general prevalence reduction by population approach. The combined approach was most effective in prolonging survival when the high risk group being targeted was either the lowest socioeconomic quintile or the mentally ill. For those in the lowest socioeconomic quintile this gain was 0.48 years for males and 0.31 years for females when smoking prevalence was reduced to 10%, which were around 49 and 35% respectively of the maximal gains in ALE that could be achieved through complete cessation using combined approach. For those who were mentally ill this gain was 0.53 years for males and 0.36 years for females which were around 51 and 42% respectively of the maximal gains in ALE that could be achieved through complete cessation using combined approach.

## Discussion

In Australia, smoking is the single most important preventable cause of ill health and death even though the prevalence of smoking has been declining since the 1950s, and Australia currently has one of the lowest rates of smoking in the developed world. Prevalence of smoking has declined more rapidly in high risk groups particularly among those belonging to socially disadvantaged groups. As a result, there is a narrowing down of gap in smoking prevalence between high risk and low risk population groups. Smoking prevalence is still double in high risk population groups which is why one of the key priority areas of Australia’s 2012–18 National Tobacco Strategy has been to strengthen efforts to reduce smoking in these groups [45]. But, not all tobacco control measures effectively used in the general population may be effective in specific high-risk population groups. Therefore, in addition to population-based strategies, another way for effectively reducing smoking in high risk population groups may be to target these groups to more effectively identify and support those smoking to quit, also known as the high risk approach. It is however unknown whether the population-based tobacco control intervention is more effective in increasing life expectancy among Australians compared to a high risk tobacco control intervention. A previous study examined gains in life expectancy in Australia due to reduction of smoking prevalence to 0 and 10% in the whole population and below different ages [46], but not for the key high risk population subgroups for reducing smoking prevalence, a task which was undertaken in the present study.

We considered all-cause mortality rather than disease specific mortality as our outcome for estimating average life expectancy because the former is an ultimate indicator of health. Using the risk percentiles approach which assumes that baseline death rates remain stable throughout lifespan and there is no net migration, analyses were performed separately for men and women using actual data from the 2014–15 NHS, simulated data from various sources and actual population and death counts for 2015–17. We examined the key high risk population subgroups such as people belonging to lowest quintiles, indigenous, homeless, prisoners and mentally ill. A 10% smoking prevalence by 2020 was proposed as a feasible target by the National Preventive Taskforce [47] and there is growing evidence that this is well underway [48]. Thus, the scenarios we analyzed are expected to be potentially achievable.

The results showed that for reduction to a smoking prevalence of 10% the population approach is more effective in terms of life expectancy gains than the high risk approach of targeting the homeless or prisoner or low SES or indigenous, but is less effective than targeting mentally ill. The latter which although represents a reasonably large fraction of smoking population doesn’t have as high smoking prevalence as some other high risk population groups examined, particularly homeless and prisoners. For complete smoking cessation, the population approach is found more effective than the high risk approach. The combined approach is however the most effective for reducing smoking to both 10 and 0% and is the optimum preventive strategy for reducing smoking prevalence.

The optimum strategy for preventing smoking-related diseases, however, depends on the disease to be prevented; the distribution of smoking prevalence in the population and the likelihood of achieving the desired reduction in smoking prevalence. The major smoking-related diseases in Australia such as CVD, lung cancer and COPD are concentrated in low or moderate risk population subgroups. For instance, AIHW (2014) reported that combinedly for the Australian states of New South Wales, Victoria, Queensland, Western Australia and that of Northern Territory, 98.05% (*n* = 42,498) of new cases of lung cancer during 2008–2012 came from the non-indigenous population which is broadly a low risk population subgroup compared to the indigenous, whereas only 1.95% (*n* = 845) arose from the latter which is a high risk population subgroup [49]. Similarly, people with cardiovascular diseases were more likely (78%) to live in a low risk population subgroup, e.g., those living in less disadvantaged socioeconomic areas, compared to those living in a high risk population subgroup, for example, those living in highly disadvantaged socioeconomic areas (22%) [50]. As non-indigenous and less socially disadvantaged people live all across Australia this may imply that lung cancer and cardiovascular diseases are widespread across Australia although in high risk groups lung cancer [51] and CVD [52] are more common in relative terms. Furthermore, despite many years of targeting high risk groups for smoking reduction, smoking prevalence in these groups still remain considerably higher than low or moderate risk groups.

In this study, the effect of smoking cessation and reduction of smoking prevalence to 10% when the whole population is targeted for such reduction was lower than that encountered in the previous Mannan et al. (2016) study [46] using the same methodology. This difference is mainly due to the decline in smoking prevalence in 2014–2015 compared to 1999–2000 which was the baseline for the previous study.

In the present analysis the absolute risk of death of individuals was estimated prospectively on the basis of baseline cardiovascular risk factors similar to a cohort study. From the positive side it reduces the time and cost associated with collecting repeated measures data for risk factors; on the contrary, the use of life table method in a cross-sectional setting makes certain assumptions for estimating the long-term effects of changes in the smoking behavior on improvements in life expectancy. The key assumption which was made is that the age-sex specific mortality rate for each risk percentile for the baseline and intervention scenarios would continue for the rest of the life of each member of a risk percentile. The EURO SCORE did not separate past smoking from never smoking. The impact of ignoring this risk stratification on life expectancy gains was not directly examined. However, since for quitting as high as before 55 years, there is no excess risk of death from all major CVD causes compared to never smokers [3], the effect of not making the above distinction in smoking categorization, on the life expectancy gains, may not be too pronounced. In this study we did not recalibrate a Framingham based all-cause mortality risk equation [53] to the Australian population, because it was not developed separately for men and women as was required for this study.

The more well-known population attributable risk method [54] is simpler than the risk percentiles method for estimating improvements in life expectancy. But, the advantage of using risk percentiles method is that it already incorporates the life table and so we can estimate life expectancy as a direct output. In contrast, the population attributable risk method alone is not sufficient to estimate improvements in life expectancy.

The non-J shaped relationship between intensity and duration of cigarette smoking and chronic diseases implies that the population approach reduces the risk of everyone in the population [55]. In Australia, the major smoking-related diseases are spread all across the population. However, since the combined approach of reducing smoking is most effective for improving life expectancy of Australians particularly when the high risk groups have a high fraction of smokers in the population, it is evident that targeting these groups for smoking reductions can strongly complement the existing population-based smoking reduction strategies. We believe that the “high risk” approach is not interfering with potential “population strategies”. Population strategies like increasing price and duty for cigarettes, bans on advertising, public health campaigns, smoke free legislation, jurisdictional bans and limitations on point of sale display, mandatory graphic health warnings on packaging will help to further reduce smoking prevalence in Australia. We believe that although the disparity in smoking prevalence between high risk and normal or low risk population subgroups has started to narrow down, this can be accelerated by specifically targeting high risk groups for smoking reduction. By applying systematic policy and population-based tobacco control programs with proven effectiveness to these high risk groups, their smoking prevalence can be reduced and subsequently tobacco-related disparities between the groups can be narrowed down. Furthermore, tailoring of population-based tobacco control strategies in these specific population groups is necessary when it’s appropriate.

## Conclusions

Targeting both the population as well as a high prevalent smoking subgroup having a large fraction of smokers in the population for smoking reduction contributes more to population health gains in Australia than targeting the population alone for smoking reduction. Therefore, as both approaches are important, the national prevention policies should make judicious use of both to maximize health gain.

## Supplementary information

**Additional file 1.**

## Data Availability

Not applicable.

## Abbreviations

AIHW: Australian Institute of Health and Welfare
ALE: Average life expectancy
CHD: Coronary Heart Disease
CVD: Cardiovascular Disease
COPD: Coronary Obstructive Pulmonary Disease
EURO SCORE: European Cardiovascular SCORE
IS: Indigenous status
NHS: National Health Survey
SES: Socioeconomic status
SEIFA: Socioeconomic Index for Disadvantage
