# Supplementary material for: Gains in life expectancy in the Australian population due to reductions in smoking: comparisons between interventions targeting the population versus interventions in a specific high risk group
Source: BMC Public Health. 2020 Sep 29;20:1478. doi: 10.1186/s12889-020-09600-w (PMC7526239; doi:10.1186/s12889-020-09600-w)
Supplement: Supplementary file 1 — Additional file 1. [file 12889_2020_9600_MOESM1_ESM.docx]

**Appendix**

**Step 1**

Calculate the underlying risks for CHD deaths and for non-CHD deaths separately for the person's age and for their age in three years time. The underlying survival probability, S_0_,is given by:

*S*_0_*(age)=*exp{-exp(-α))(*age*)^p^}

*S*_0_*(age+3)=*exp{-exp(-α))(*age+3*)^p^} (1)

where α and p are the Weibull parameters which for females were found to be 28.7 and 6.23 for CHD deaths, 30 and 6.42 for non-CHD deaths and for males were found to be 21 and 4.62 for CHD deaths, 25.7 and 5.47 for non-CHD deaths.

**Step 2**

Calculate the weighted sum, *w*, of the risk factors cholesterol, smoking and systolic blood pressure (SBP), separately for CHD deaths and for non-CHD deaths. Smoking is coded as 1 for current smoker and 0 for non-smoker, so no value for smoking has to be entered if the person is a non-smoker. Cholesterol is measured in mmol/L and SBP is measured in mmHg. The weighted sum for CHD or non-CHD is,

w=β_cholesterol_*(cholesterol-6)+β_SBP_*(SBP-120)+β_current_ _smoker_*current smoker (2)

where β_cholesterol_, β_SBP and_ β_current_ _smoker_ are the regression coefficients for total cholesterol, SBP and current smoker when the outcome is the risk of CHD or non-CHD. These regression coefficients were estimated from the 2014-15 NHS linked to deaths registry till 2017.

**Step 3**

Combine the underlying risks for CHD and for non-CHD, at the person's age and at their age three years from now (four calculations) which were calculated in step 1 with the weighted sum of a person's risk factors from step 2 for the two end-points, CHD and non-CHD to get the probability of survival at each age for each cause.

*S(age)=S*_0_*(age)*^exp(^*^w^*^)^

*S(age+3)=S*_0_*(age+3)*^exp(^*^w^*^)^ (3)

**Step 4**

For each cause, calculate the 3-year survival probability based on the survival probability for the person's current age and their age in 3 years time:

*S*_3_(*age)=S(age+3*)/*S(age)* (4)

**Step 5**

Calculate the 3 year risk for each end-point as

Risk_3_=1-S_3_(age) (5)

**Step 6**

Combine the risks for CHD and non-CHD by adding them:

*CVD Risk*_3_(*age)=CHD Risk(age)+ Non*-*CHD Risk(age)*  (6)

**APPENDIX 1**

**The risk percentiles method**

The risk percentiles method is useful for projecting the lifetime risk of all-cause mortality and derives an average life expectancy at each percentile of projected mortality risk within a population. The aim of this model is to distribute the absolute short-term mortality risk within the population, projected using an existing all-cause mortality risk prediction equation.

The key modelling assumption is that the observed deaths will follow the same distribution as the projected risk of death and so we can use the latter distribution to allocate observed deaths to different percentiles of risk. Based on these estimated risk percentiles we can construct separate life tables for people at different levels of mortality risk and then generate risk-specific survival curves from each of these life tables.

**Stages of the risk percentiles method**

The method proceeded in two stages. In this section we outline the general principles, with details to follow in the next section. In the first stage, the observed deaths were allocated to percentiles of mortality risk. To achieve this, the EURO SCORE equation was applied to the study cohort to project the levels of risk that divide the population into percentiles of mortality risk. The overall population was evenly spread between the percentiles of mortality risk; however, the absolute risk was higher in higher percentiles and vice versa. The observed counts of deaths were then allocated to each percentile of mortality risk in proportion to the modelled level of absolute risk in each percentile, enabling mortality rates to be calculated for each percentile of risk. These mortality risks were then used to calculate life tables for each percentile of risk.

The second stage involved modelling the effect of a reduction in cigarette smoking on all-cause-mortality using the EURO SCORE equation. For example, to achieve an overall smoking prevalence of 10%, the smoking status of some AUSDIAB participants who were current smokers at the baseline examination was randomly changed to “non-smoker”, while the smoking status of all non-smokers was not changed. The revised smoking status was then used in the EURO SCORE equation along with other risk factors whose distribution remained unchanged. For a person whose status changed from smoker to non-smoker, this gave a reduced risk of mortality. This created a simulated sample of people with reduced levels of smoking prevalence compared to the observed data on smoking prevalence in the AUSDIAB baseline sample. The random selection process was replicated 1000 times and results presented are averages over the 1000 replications. An identical method was performed to achieve a smoking prevalence of 10% under a specific high risk or combined approach for reducing smoking by randomly selecting some of the current smokers pertaining to a high risk or a combined approach group at the baseline examination, to have their smoking status changed to non-smoking. To achieve a 0% prevalence scenario, all persons at the baseline examination who were smokers in the sample or in a high risk group or in a combined approach group, had their smoking status changed to non-smokers.

For each smoking reduction scenario, the participants who moved to a non-smoker category were then re-allocated to lower risk percentiles based on their new smoking status. When this was weighted using the AUSDIAB survey weights, it provided a modelled estimate of the shift in the total Australian population between each percentile group when current smokers were changed to non-smokers.

**Details of the calculation of Average life expectancy (ALE) per person**

The overall outcome measure used in this model was the average potential years of life () per person. This quantity is the projected total life years for each person belonging to an age-sex percentile group at the time of the baseline examination. The projected total life years in each risk percentile for each age-sex group are the product of the life expectancy in that group by the corresponding 2006 population count. Life expectancy is defined as the average number of years of life remaining at current age for a person. The life expectancy in each risk percentile group was calculated for each age-sex group using the life tables derived in stage 1 of the model. The total life years was summed over the percentiles and age groups within each sex group and divided by the population count to calculate a baseline per person for each sex group. Similar values were calculated for each scenario where the smoking prevalence was reduced for a specific subgroup of the population. These per person under the various smoking prevalence reduction scenarios are called the “Scenario” in the following sections.

The application of each of these model steps is described in detail below.

**Details of Stage 1: Allocating deaths to percentiles of risk**

Stage 1 of the risk percentiles model was used to derive an average life expectancy at each percentile of projected mortality risk based on the all-cause mortality equation. The steps involved in the calculation, within each age and sex group, are described below:

STEP 1: Divide the Australian population into mortality risk percentiles.

The EURO SCORE all-cause mortality equation was applied to the study cohort to calculate the five-year probability of death or the risk score for each individual. The values of the risk score which divide the cohort into percentiles were then calculated. Each cohort member was allocated to a risk percentile using their risk score and a total risk score calculated for each percentile by summing the individual risk scores for the survey participants in that percentile.

STEP 2: Use the ratios of the risk scores to allocate deaths in the Australian population to risk percentiles

The national count of deaths summed across 2013 to 2017 was allocated to the risk percentiles using the ratios of the aggregate risk score between percentiles as follows. The relative mortality risks were calculated as the ratio of the total risk scores between percentiles and the observed deaths were allocated to the percentiles according to these ratios. For example, if one percentile group had a risk score twice that of another group then the deaths were allocated between them in the ratio 2:1. When divided by the population count (assumed evenly spread between risk percentiles) this gives us the mortality rate for each risk percentile group within each age and sex group.

Calculations of the proportion of deaths in each percentile group were based on relatively small numbers in some age groups (particularly the older age groups) and so the resulting proportions were subject to some variability. To overcome this, we applied a LOESS non-parametric smoothing procedure (Cleveland et al., 1988) to the estimated proportions using the LOESS procedure in the SAS statistical software package (Cary, NC, 2014).

STEP 3: Use these mortality rates to construct sex-specific life tables for each risk percentile within each sex group

After step 2, we had a set of age specific mortality rates by sex for each percentile group. We applied standard life table techniques (Chiang, 1984) using these mortality rates to construct a sex-specific life table for each percentile group. These life tables were used to generate sex-specific life expectancies for each percentile group.

STEP 4: Calculate a baseline average life expectancy (ALE) per person for each sex

The average life expectancy by sex for each percentile group was extracted from the life tables calculated in step 3 above. This life expectancy was multiplied by the corresponding 2006 population count to get the projected total life years by sex for each percentile group. This was summed over the percentiles and ages and divided by the population count to calculate a baseline ALE per person by sex.

STEP 5: Use bootstrapping to construct confidence intervals for ALE

Estimation of confidence intervals for ALE derived from life tables can be performed with closed form equations, however because the major uncertainty in any modelled value arises from the uncertainties inherent in the modelling process, these confidence intervals are not appropriate in the current context. Instead a measure of the variability in the ALE was derived by applying nonparametric bootstrapping techniques to the study cohort from which the estimates were derived.

The above calculations were done at the decile risk level.

**Details of Stage 2: Modelling the effect of Reductions in smoking prevalence on mortality**

As noted above, the effect of intervening to reduce smoking prevalence on mortality was modelled for the 2006 Australian population. The detailed steps involved in the calculation were as follows:

STEP 1: Change a certain percentage of current smokers to non-smokers.

The risk factors modelled in this paper were those in the EURO SCORE equation which are potentially modifiable—smoking, total cholesterol and systolic blood pressure (SBP) (Conroy et al., 2003). We returned to the study cohort to model the effect of changing specific percentages of current smokers to non-smokers. For example, in examining the effect of a reduction in smoking prevalence a certain percentage of cohort members who were current smokers had their risk scores recalculated assuming that their smoking status changed to a non-smoker.

STEP 2: Re-allocate participants to risk percentiles

Those who had their risk scores reduced in the simulated sample because of being categorized as a non-smoker instead of a smoker were then re-allocated to a lower risk percentile group on the basis of their revised risk score but using the existing risk score cut-offs as calculated in Step 1 of stage 1. Note that this implies that the ‘percentile’ groups in the simulated sample are no longer true percentiles. The participants who had their risk scores reduced moved to a lower ‘percentile’ group. Hence the participants in the simulated sample were no longer evenly distributed between the groups. So in the description of stage 2 of the modelling we will refer to these groups as the risk groups.

This re-allocation, provided a modelled estimate of the shift in the study cohort between each risk group which would arise if all high risk categories of a risk factor in the cohort were reduced to the target category. For example, in examining a reduction in smoking prevalence, our simulated sample contained certain percentage of participants whose true smoking status was current smoker and whose simulated smoking status was non-smoker. Each of these participants was re-allocated to a lower risk group by re-calculating their risk score using the simulated smoking status category of non-smoker and allocating them to a new risk group using this re-calculated risk score.

STEP 3: Calculating the scenario ALE

We have a projected average life expectancy by sex and age for each risk group calculated from step 4 of stage 1. We applied the modelled shift from Step 2 above to the 2017 Australian population counts to derive a modelled estimate of the population by age and sex for each risk group. We multiplied the average life expectancy for each percentile-age-sex group by the new population count projected to be in that group under each scenario. This was summed over the percentiles and ages and divided by the population count to calculate a scenario ALE.

For example, we calculated the altered smoking prevalence scenario ALE by projecting the population shift between risk groups associated with converting certain percentage of current smokers into non-smokers. We then multiplied the average life expectancy in each risk group for each age-sex by the new population count projected to be in that group after certain percentage of current smokers had become non-smokers.

STEP 4: Projecting the impact of each scenario intervention

The impact of each scenario intervention was measured by comparing the baseline ALE per person with the scenario ALE per person. For example, the impact of reduction in smoking prevalence was measured by comparing the baseline ALE per person with the declining smoking prevalence scenario ALE per person described in step 3 above. This was repeated for the 1000 simulation replications and results averaged across replications. The gain in ALE per person due to the intervention in a particular scenario is then the scenario APYL minus the baseline ALE per person.

STEP 5: Use bootstrapping to construct confidence intervals of scenario ALE

To obtain confidence interval of a scenario ALE and gain in ALE taking into account all forms of uncertainty we applied nonparametric bootstrapping techniques [21] to the AUSDIAB sample from which the estimate was derived.

**A SIMPLE ILLUSTRATION OF THE PROCEDURE FOR CALCULATING ALE**

The procedure for calculating ALE is illustrated in Table S1 with a hypothetical population and intervention on 100,000 people divided into risk quartiles (not percentiles) for ease of presentation. Column 2 presents the number of people in each quartile at baseline and column 3 is a hypothetical average life expectancy for each quartile. Column 4 presents the baseline life-years estimate, which is calculated by multiplying column 2 by column 3. If we sum column 4 and divide this by the total population, we get the baseline ALE per person (32.5 years). Column 5 presents the number of people allocated to each risk group after the survey participants have had their risk adjusted according to the smoking cessation target (0% or 10%). Column 6 represents the intervention life year estimate and is calculated by multiplying column 3 by column 5. If we sum column 6 and divide this by the total population, we get the intervention ALE (35.0 years). Therefore, the gain in ALE across the population due to the intervention is 35.0-32.5 = 2.5 years.

Table S1 Modelling the impact of a risk factor illustrated with a hypothetical population

| **Quartile of risk** | **Baseline population distribution** | **Average life expectancy** | **Total Baseline ALE in each quartile** | **Intervention population distribution** | **Total Intervention ALE in each quartile** |
| --- | --- | --- | --- | --- | --- |
| **1** | 25,000 | 40 | 1,000,000 | 40,000 | 1,600,000 |
| **2** | 25,000 | 35 | 875,000 | 30,000 | 1,050,000 |
| **3** | 25,000 | 30 | 750,000 | 20,000 | 600,000 |
| **4** | 25,000 | 25 | 625,000 | 10,000 | 250,000 |
| **Total** | 100,000 |  | 3,000 | 100,000 | 3,500,000 |
| **Overall APYL** |  |  | 32.5 |  | 35.0 |

cALCULATION OF GAIN IN ALE PER PERSON AMONG SMOKERS IN THE POPULATION

The APYL per person calculated using the above approach is the average over the entire population, which contains smokers and non-smokers, with weighting in proportion to the prevalence of smoking. Similarly for the gain in ALE for a specific scenario compared to the baseline scenario.

The APYL per person calculated using the above approach is the average over the entire population, which contains smokers and non-smokers, with weighting in proportion to the prevalence of smoking. Similarly, for the gain in ALE for a specific scenario compared to the baseline scenario.
